# TRP channels in mammalian hearing loss

**DOI:** 10.3389/fnmol.2025.1626640

**Published:** 2025-07-04

**Authors:** Zhidong Zhang, Baoshan Wang

**Affiliations:** Department of Otolaryngology-Head and Neck Surgery, The Second Hospital of Hebei Medical University, Shijiazhuang, China

**Keywords:** TRP channels, hearing loss, cochlea, sensory transduction, therapeutic targets

## Abstract

Hearing loss, a common sensory disorder, significantly diminishes quality of life and can stem from diverse causes, including genetic predispositions, aging, noise exposure, and ototoxic medications. Recent research has highlighted the involvement of transient receptor potential (TRP) channels in auditory function and hearing loss. This review offers a comprehensive overview of the current knowledge regarding the roles of TRP channels in mammalian auditory function and hearing loss. By synthesizing the latest research findings, this review aims to elucidate the complex interplay between TRP channels and auditory function, emphasizing their significance in the pathogenesis of hearing loss and identifying potential targets for future therapeutic interventions.

## 1 Introduction

In recent years, transient receptor potential (TRP) channels have emerged as pivotal players in auditory research, primarily due to their involvement in a wide range of sensory responses, including heat, cold, pain, stress, vision, taste, and mechanosensation (Caterina et al., 1997; Tominaga and Julius, 2000; McKemy et al., 2002; Christensen and Corey, 2007; Ishimaru and Matsunami, 2009; Katz et al., 2017).

These channels are permeable to various cations, including calcium, sodium, potassium, and magnesium (Tsagareli and Nozadze, 2020), positioning them as key components in cellular signaling pathways. Many TRP channels are expressed in the cochlea, the organ responsible for hearing. This widespread expression suggests that TRP channels may significantly influence auditory function and contribute to hearing loss.

Recent studies have implicated TRP channels in multiple types of hearing loss, including drug-induced, noise-induced, age-related, and genetic hearing loss. Despite increasing research attention, a comprehensive understanding of TRP channels' roles and mechanisms in hearing loss remains fragmented.

This review aims to provide an in-depth overview of the current knowledge regarding TRP channels' involvement in auditory function and hearing loss. We will discuss the specific roles of different TRP channel subfamilies in auditory function and hearing loss, and their potential as therapeutic targets. By synthesizing existing literature and integrating findings from various studies, we hope to elucidate the complex interplay between TRP channels and hearing loss, offering insights into the molecular mechanisms underlying hearing-related disorders and identifying potential therapeutic targets for treating hearing loss.

### 1.1 Classification and general structure of TRP channels

Since the first TRP channel was discovered in a mutant strain of *Drosophila melanogaster* by Cosens and Manning (1969) and later identified by Montell and Rubin (1989). The search for TRP homologs in mammalian species has, to date, revealed 28 TRP channels. These channels are classified into seven subfamilies: TRPA (Ankyrin), TRPC (Canonical), TRPM (Melastatin), TRPML (Mucolipin), TRPP (Polycystin), TRPV (Vanilloid), and TRPN (NOMPC) based on sequence and topological differences (Hardie, 2011). In mammals, TRPN(NOMPC) proteins are not found, although they are expressed in some vertebrates, such as zebrafish (Montell et al., 2002; Corey, 2003; Clapham, 2003; Venkatachalam and Montell, 2007b). These TRP channels contain different members as shown in Table 1. In humans, only six TRPC channels are expressed due to the fact that human TRPC2 is a pseudogene (Wes et al., 1995).

**Table 1:** TRP channels involved in mammalian hearing loss.

**TRP channel type**	**Hearing disorders**	**References**
TRPV1	Aminoglycoside-induced hearing loss, cisplatin-induced hearing loss, noise-induced hearing loss, age-related hearing loss	Lee et al., 2013; Jiang et al., 2019; Mukherjea et al., 2011; Bhatta et al., 2019; Bauer et al., 2007; Dhukhwa et al., 2019; Zhang et al., 2023
TRPV3	Hearing impairment (about 27.7% of 2–3-month-old TRPV3 knockout mice developed hearing impairment), compensatory upregulation of TRPV4	Wang et al., 2019; Ishibashi et al., 2008, 2009
TRPV4	Increased vulnerability to acoustic injury, delayed-onset hearing loss, involved in gentamicin-induced hearing loss	Tabuchi et al., 2005; Ishibashi et al., 2008; Lee et al., 2013; Wang et al., 2019.
TRPV5	Age-related hearing loss (regulated by klotho protein)	Takumida et al., 2009; Yamauchi et al., 2010
TRPV6	Age-related hearing loss (calcium dyshomeostasis)	Takumida et al., 2009; Morgan et al., 2018
TRPM1	None (TRPM1 knockout mice showed normal hearing and balance)	Gerka-Stuyt et al., 2013; Bellone et al., 2008
TRPM2	Unclear	Bal et al., 2020
TRPM4	Likely involved in auditory system maturation and maintenance of inner ear function	Sakuraba et al., 2014
TRPM6, TRPM7	Conditional knockout mice showed no auditory deficits	Morgan et al., 2018; Zou et al., 2019
TRPML1, TRPML3	TRPML3 gain-of-function mutations cause hearing loss; combined TRPML1/TRPML3 knockout accelerates age-related hearing loss	Palma et al., 2002; Nagata et al., 2008; Takumida and Anniko, 2010; Grimm et al., 2007; Wiwatpanit et al., 2018
TRPC3	Enhanced high-frequency hearing sensitivity in knockout mice; multi-knockout causes hearing loss and vestibular deficits	Phan et al., 2010; Wong et al., 2013; Quick et al., 2012; Sexton et al., 2016
TRPC6	Combined TRPC3/TRPC6 knockout leads to hearing loss; multi-knockout elevates high-frequency thresholds	Englisch et al., 2023; Quick et al., 2012; Sexton et al., 2016
TRPA1	Modulates cochlear sensitivity post-noise exposure (knockout mice show transient threshold shifts and ABR latency changes)	Corey et al., 2004; Kwan et al., 2006; Vélez-Ortega et al., 2023

**Figure 1:**
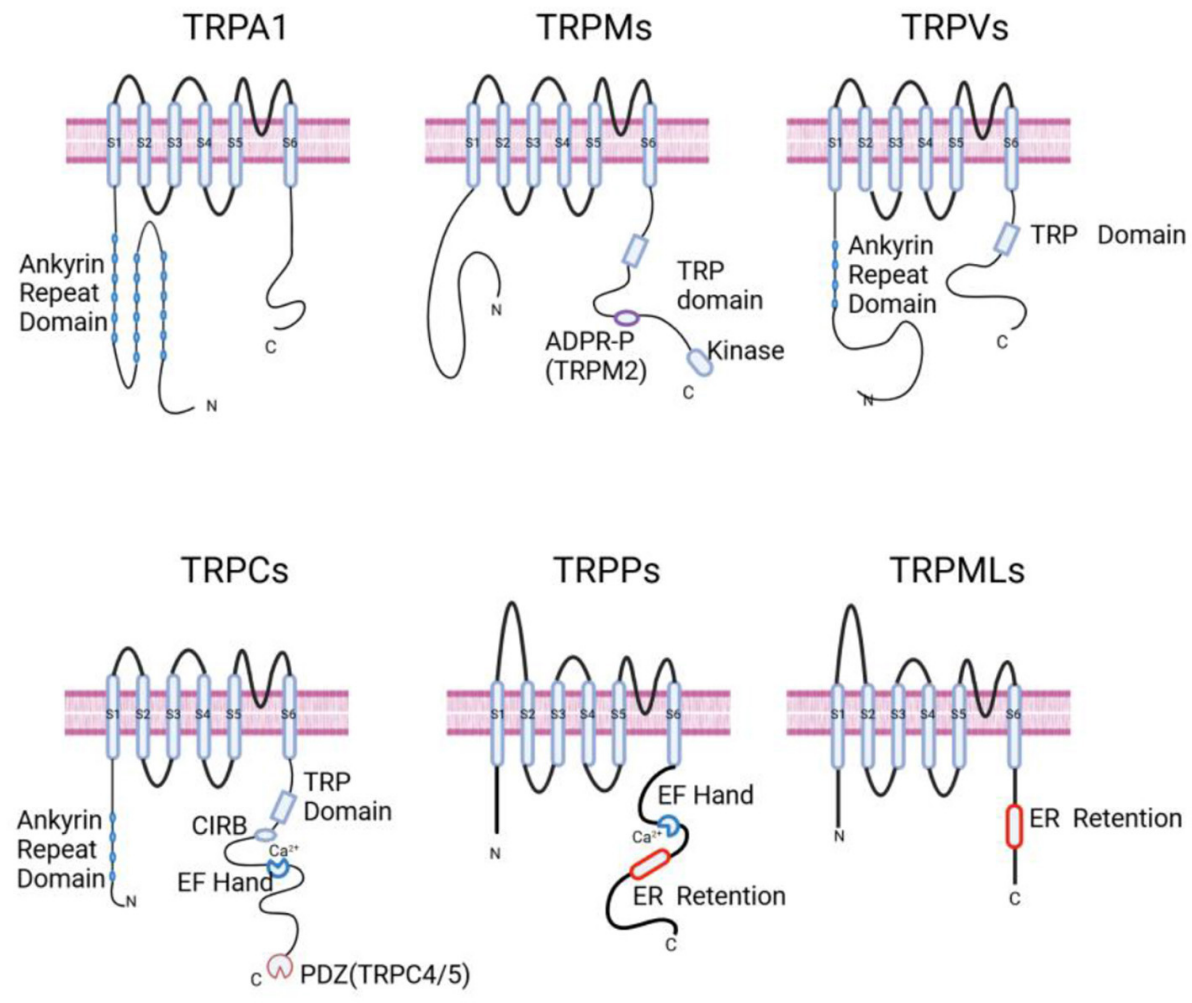
Schematic representation of the general structures of TRP channels. TRPA1: displays an Ankyrin Repeat Domain at the N-terminus. Ankyrin Repeat Domain is a motif composed of two helices that are separated by loops; TRPMs: characterized by the presence of a TRP domain and an ADPR-P binding site specific to TRPM2, along with a Kinase domain at the C-terminus. TRP Domain is a conserved region present in the TRPV, TRPM, and TRPC families; ADPR-P (ADP-ribose pyrophosphatase) domain is a homologous region in TRPM2 that functions as a phosphohydrolase and binds ADP-ribose; TRPVs: features an Ankyrin Repeat Domain and a TRP Domain. TRPCs: contains an Ankyrin Repeat Domain, a CIRB Domain, a TRP Domain, an EF Hand for calcium binding, and a PDZ motif specific to TRPC4 and TRPC5. CIRB Domain is a calmodulin/IP3 R-binding (CIRB) domain; EF Hand Domain has the ability to bind Ca^2+^; ER Retention Domain is a small domain that presumably keeps the channel localized in the endoplasmic reticulum; PDZ (postsynaptic density 95/disc-large/zona occludens) Domain is a typical protein interaction motif that helps form signaling complexes. TRPPs: show an EF Hand for calcium binding and an ER Retention motif; TRPMLs: have an ER Retention domain at the C-terminus. Created in BioRender. Zhang (2025) https://BioRender.com/dnjphq7.

TRP channels typically comprise six transmembrane-spanning domains (S1–S6). A pore-forming loop, located between S5 and S6, mainly controls the passage of cations through the channel. Moreover, both the N-terminus and C-terminus of the channel are located within the intracellular spacewith different functional domains. As presented in Figure 1, different TRP channels possess characteristic domains in either the N-terminal or C-terminal regions. These specific domains are closely associated with their functional and regulatory characteristics (Wu et al., 2010; Venkatachalam and Montell, 2007a).

**Figure 2:**
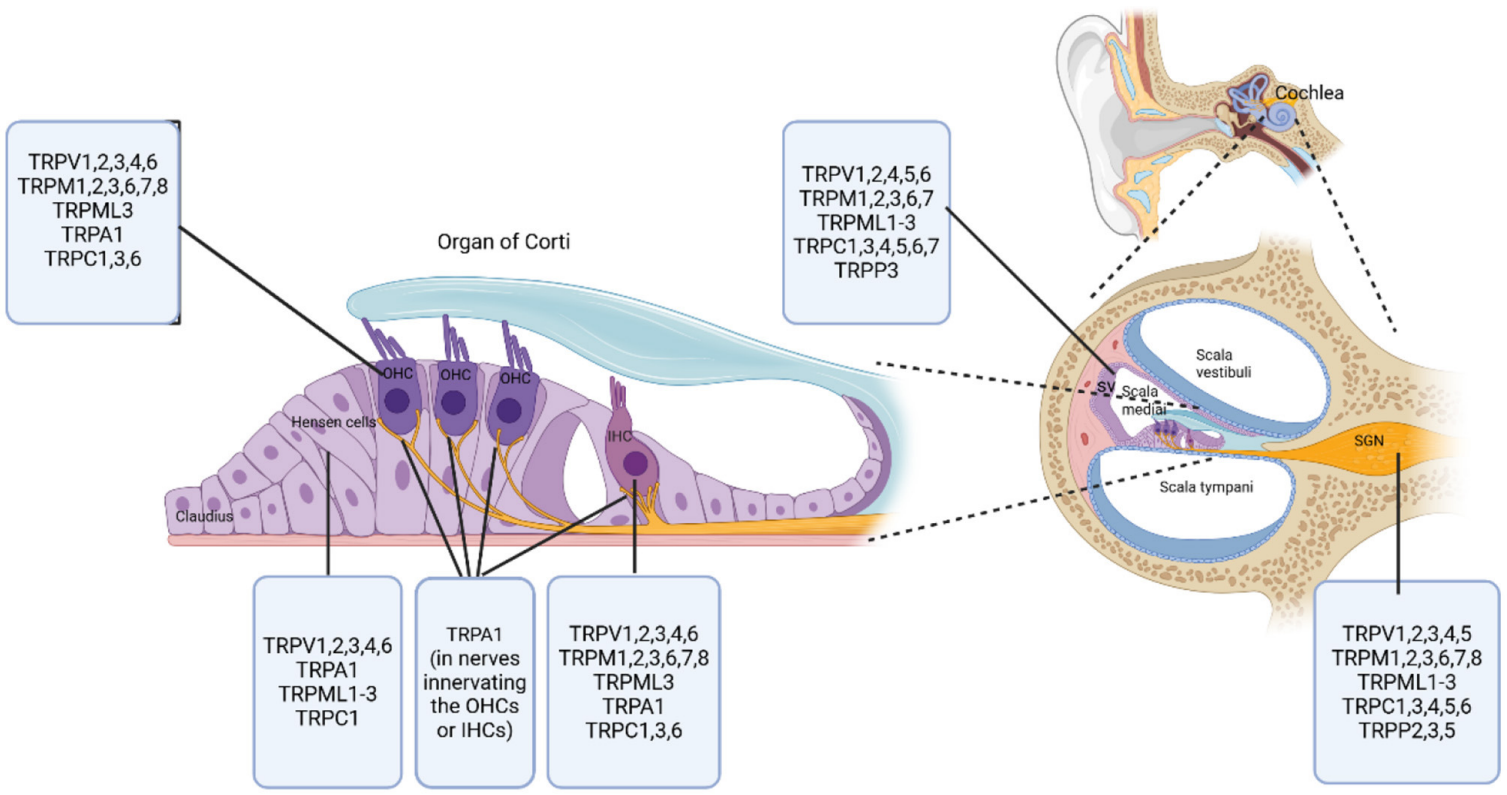
Distribution of TRP channel subfamilies in the mammalian cochlea. This diagram illustrates the specific locations and distributions of various TRP channel subfamilies within the mammalian cochlea. The right side of the diagram shows a cross-sectional overview of the cochlear compartments, with labeled compartments including Scala vestibuli, Scala media, Scala tympani, Stria vascularis (SV), and Spiral ganglion (SGN). At the center of the diagram is the organ of Corti, highlighting key cell types including outer hair cells (OHCs), inner hair cells (IHCs), Hensen cells, and Claudius cells. Created in BioRender. Zhang (2025) https://BioRender.com/aikl15h.

TRP channels are extensively expressed in the inner ear of mammalian species (Figure 2). This suggests their crucial role in mammalian auditory function.

## 2 TRPV and hearing function

The TRPV subfamily can be further divided into six members, namely TRPV1-6. Members of the TRPV subfamily are characterized by their sensitivity to diverse stimuli, including temperature, pH, and specific chemical ligands (Pumroy et al., 2020; Zhang et al., 2021). These channels function as nonselective cation channels, with permeability to both calcium and sodium ions (Brito et al., 2014). TRPV family members are extensively expressed in spiral ganglion neurons, hair cells and stria vascularis of the cochlea (Ishibashi et al., 2008; Yamauchi et al., 2010; Wang et al., 2019). Their wide distribution indicates that TRPV channels play a crucial role in auditory function and are associated with various types of hearing loss.

### 2.1 TRPV1 and hearing loss

#### 2.1.1 TRPV1 and drug-induced hearing loss

##### 2.1.1.1 TRPV1 and aminoglycoside-induced hearing loss

The Transient Receptor Potential Vanilloid 1 (TRPV1) is a non-selective cation channel that was first cloned in 1997 (Caterina et al., 1997). TRPV1 can be activated by various exogenous or endogenous stimuli, which cause the channel to open and allow the influx of calcium ions (Tóth et al., 2004). Exogenous agonists of TRPV1 include heat, protons, and capsaicin, making it a multimodal sensor for noxious stimuli. Endogenous agonists encompass products of lipid metabolism, hormones, neurotransmitters, and gas molecules such as hydrogen sulfide (Benítez-Angeles et al., 2020). The activation and upregulation of TRPV1 have been closely linked to hearing loss.

Systemic use of aminoglycosides is a well-recognized cause of ototoxicity, with the drug being taken up in the cochlea and leading to hair cell damage. The multiple mechanisms by which hair cells can ingest aminoglycosides, such as endocytosis, transporters, and ion channels, highlight the complexity of this process (Jiang et al., 2017). The identification of TRPV1 as a crucial mediator in the uptake of aminoglycosides by hair cells is a significant finding. The study using cochlear explants from Sprague-Dawley rats clearly demonstrated this role for TRPV1 in gentamicin uptake (Lee et al., 2013). They observed that desensitization of the TRPV1 channel by high extracellular calcium concentrations or inhibition of TPRV1 can mitigate TRPV1-mediated gentamicin uptake (Mandadi et al., 2004). This not only provides a specific molecular target for further investigation but also offers potential avenues for intervention.

The involvement of TRPV1 in gentamicin uptake in immature spiral neurons is yet another area of focus. Notably, knocking down TRPV1 in neonatal mouse spiral neurons significantly reduces gentamicin uptake. Additionally, treating P7 mouse cochlear explants with the TRPV1 inhibitor AMG-517 effectively diminishes the production of reactive oxygen species and apoptosis induced by aminoglycoside toxicity. These findings hold great promise for further research and potential therapeutic applications. However, the observation that intraperitoneal injection of 1 mg/kg AMG-517 failed to improve hearing in adult mice with aminoglycoside-induced hearing loss suggests that the efficacy of targeting TRPV1 may be age-dependent (Bai et al., 2023). This age-related difference in the response to TRPV1 inhibition is an important consideration for future research. It may suggest that different strategies need to be developed for preventing or treating aminoglycoside-induced hearing loss in different age groups.

The discovery that systemic inflammation can upregulate TRPV1 expression in outer hair cells of mice, thus promoting gentamicin uptake adds another layer of complexity (Jiang et al., 2019). Specifically, lipopolysaccharides (LPS) exposure significantly increased TRPV1 expression in the cochlea, particularly in outer hair cells (OHCs), and enhanced gentamicin uptake in these cells. In contrast, TRPV1 knockout mice showed reduced gentamicin uptake and less severe hearing loss under inflammatory conditions. It shows how external factors like inflammation can interact with the molecular mechanisms of ototoxicity and highlights the critical role of TRPV1 in mediating inflammation-induced ototoxicity. Despite this, the involvement of inflammation in cisplatin-induced hearing loss was also found is closely related with TRPV1, as discussed in the following subsection.

##### 2.1.1.2 TRPV1 and cisplatin-induced hearing loss

Cisplatin is a widely used and highly effective chemotherapy drug for treating various solid tumors. However, its clinical application is often limited by its ototoxicity, which can lead to irreversible hearing loss in patients during treatment (Kros and Steyger, 2019). This side effect poses a significant challenge in balancing the therapeutic benefits of cisplatin with its potential harm to hearing. Recent studies have shed light on the role of TRPV1 in cisplatin-induced ototoxicity. It has been demonstrated that TRPV1 is upregulated in the cochlea of rats following cisplatin exposure (Mukherjea et al., 2008). Inhibition of TRPV1 in UB/OC-1 cell cultures can reduce TRPV1 expression and mitigate the damage to these cultures. Furthermore, local application of TRPV1 siRNA via the round window in rats has been shown to reduce cisplatin-induced hearing loss (Mukherjea et al., 2008). This suggests that targeting TRPV1 could be a potential therapeutic strategy to protect against cisplatin ototoxicity.

Another important player in this process is NADPH Oxidase 3 (NOX3). Knockdown of NOX3 using siRNA has been found to reduce TRPV1 expression and protect against cochlear damage and hearing loss (Mukherjea et al., 2010). This finding highlights the interplay between NOX3 and TRPV1 in mediating cisplatin-induced ototoxicity. The link between these two proteins provides a promising avenue for developing novel treatments to mitigate the ototoxic effects of cisplatin.

Interestingly, capsaicin, a TRPV1 agonist, has been shown to have a dual role in cisplatin-induced hearing loss. On one hand, capsaicin-mediated activation of TRPV1 can induce an inflammatory process thus exacerbating cisplatin-induced damage to hair cells (Mukherjea et al., 2011). This process involves the activation of NOX3 and the subsequent generation of reactive oxygen species (ROS). The study showed that capsaicin-induced activation of TRPV1 leads to increased NOX3 activity, which in turn generates ROS. These ROS then activate Signal Transducer and Activator of Transcription 1 (STAT1), a key regulator of inflammatory gene expression. The activation of STAT1 results in the upregulation of pro-inflammatory cytokines such as TNF-α, iNOS, and COX-2, which contribute to the inflammatory response in the cochlea. On the other hand, capsaicin can also protect against cisplatin-induced ototoxicity by binding to CB2 (Cannabinoid 2) receptors and activating STAT3, which is known to have anti-inflammatory and cell survival-promoting effects (Bhatta et al., 2019). This dual role of capsaicin is intriguing and suggests a complex interplay of signaling pathways in determining its effects on hearing. The dual role of capsaicin in cisplatin-induced ototoxicity underscores the need for further research to elucidate the precise molecular mechanisms at play.

Future studies should focus on clarifying the factors that determine whether capsaicin exacerbates or protects against cisplatin-induced hearing loss. Additionally, exploring the potential therapeutic applications of targeting TRPV1 and NOX3, as well as understanding the role of capsaicin in these pathways, could lead to the development of novel strategies to mitigate the ototoxic side effects of cisplatin while preserving its anticancer efficacy.

#### 2.1.2 TRPV1 and noise-induced hearing loss

In addition to drug-induced hearing loss, noise-induced hearing loss is also associated with TRPV1. Following noise exposure, the level of TRPV1 in spiral ganglion neurons of rats increased greatly (Bauer et al., 2007). This initial finding highlights TRPV1 as a potential key player in the cochlear response to noise trauma. The study by Dhukhwa et al. (2019) further elaborated on this relationship. They demonstrated that noise trauma not only increases the level of TRPV1 but also the TNF-α mRNA in the rat cochlear. The increased TRPV1 was found to result in an increase in calcium entry, and significantly, this process is potentiated by TNF-α. These results show a connection between TRPV1 upregulation and the activation of an inflammatory pathway, as TNF-α is a well-known inflammatory cytokine. In their experiment, desensitizing TRPV1 with a desensitizing dose of capsaicin (Oral, 20 mg/kg, Wistar rats), can protect against elevated auditory thresholds caused by noise exposure in rats and downregulate the expression of the inflammatory factor TNF-α, indicates that targeting TRPV1 could be a promising strategy for preventing or treating noise—induced hearing loss.

#### 2.1.3 TRPV1 and age-related hearing loss

Building on the established associations between TRPV1 and drug/noise-induced hearing loss, research into the role of TRPV1 in age-related hearing loss (ARHL) has yielded significant insights through the use of animal models and investigations into dietary and genetic-metabolic interactions.

Studies involving TRPV1 mutant mice have provided valuable information. These mice display normal hearing sensitivity during their young adult phase. However, as they age, they develop high-frequency hearing loss (Li, 2016). This observation strongly implies that TRPV1 likely plays a protective role in safeguarding auditory function as the body ages. The progressive deterioration of hearing sensitivity in TRPV1 mutant mice indicates that the absence of this channel exacerbates the age-related degradation of cochlear structures.

Dietary factors, particularly high-fat diets (HFD), have also emerged as important players in the development of ARHL. In a study on C57BL/6J (B6) and CBA/CaJ (CBA) mice, interesting differences were observed (Zhang et al., 2023). B6 mice fed a HFD demonstrated reduced age-related hearing loss compared to CBA mice on a normal diet. The B6 mice on HFD showed better preservation of Prestin levels and outer hair cell (OHC) morphology. Prestin is a key protein involved in OHC electromotility, which is essential for cochlear amplification and frequency selectivity. The maintenance of Prestin levels and OHC morphology is indicative of better-preserved cochlear function (Dallos et al., 2008). Further analysis revealed that in B6 mice on HFD, the activation of TRPV1 and downregulation of arachidonic acid led to a suppression of the inflammatory response, thereby protecting the cochlea from ARHL. This finding is remarkable as it uncovers a complex interplay between diet, TRPV1 regulation, lipid metabolism (as arachidonic acid is a lipid-derived molecule), and inflammation in the context of ARHL. It suggests that TRPV1 expression and lipid homeostasis are intricately regulated by genetic and metabolic factors, and these regulatory mechanisms have a profound impact on cochlear function and the progression of ARHL. Overall, these studies highlight the multi-faceted nature of ARHL and the potential of targeting TRPV1 and related pathways, along with dietary modifications, as therapeutic strategies for this prevalent condition.

In summary, TRPV1 plays a crucial role in various types of hearing loss, with its activation and up-regulation closely associated with inflammatory responses. In aminoglycoside-induced hearing loss, TRPV1 mediates drug uptake, and inflammation exacerbates this toxicity by up-regulating TRPV1. In cisplatin-induced hearing loss, TRPV1 activation triggers inflammation via NOX3 and STAT1, further damaging hair cells. Additionally, the up-regulation of TRPV1 following noise exposure is linked to increased levels of the inflammatory cytokine TNF-α, indicating its role in noise-induced hearing loss. These studies demonstrate that TRPV1 is not only a key channel for drug and noise uptake but also an important mediator of inflammatory responses, offering potential therapeutic targets for the prevention and treatment of hearing loss.

#### 2.1.4 TRPV1 as a potential target for the treatment of sensorineural hearing loss

Drug development centered around TRPV1 channels has predominantly been directed toward pain management and the treatment of neurological disorders (Brito et al., 2014). Regarding hearing loss, TRPV1 channel-related targets might potentially present novel therapeutic approaches for the prevention and treatment of drug-induced, noise-induced, and age-related hearing loss. A number of Traditional Oriental Medicine (TOM) herbs show potential in treating sensory-induced hearing loss, though the exact mechanisms are unclear. Some may act via TRPV1 (Castañeda et al., 2019). For instance, puerarin, a major traditional Chinese medicine component, has anti-oxidative and anti-inflammatory properties (Wang et al., 2022). It may mitigate cisplatin-induced hearing loss by modulating TRPV1 in cochlear hair cells (Lin et al., 2024). TS, a combination of Cuscutae Semen and Rehmanniae Radix Preparataan, its active ingredients including hyperoside, quercitrin, and kaempferol, can reduce drug and noise-induced hearing loss, potentially by downregulating TRPV1 in HEI-OC1 cells (Hong et al., 2023). Pipratine, with anti-tumor properties, may treat aminoglycoside-induced hearing loss by downregulating cochlear TRPV1 and activating the PI3K-AKT pathway (Zallocchi et al., 2024). Despite these promising results, more research on TRPV1-targeting drugs is expected.

### 2.2 TRPV3, TRPV4, and hearing loss

TRPV3 and TRPV4 are mechanosensitive, non-selective cation channels that are activated by a variety of stimuli, such as temperature, osmotic pressure, and ligands (Mutai and Heller, 2003; Su et al., 2023).

#### 2.2.1 Protective role of TRPV3 in auditory function and its compensatory mechanisms

In mice, TRPV3 is expressed in inner hair cells (IHCs), yet its expression is predominantly observed in cochlear OHCs. Notably, TRPV3 expression is confined to the cytoplasm and is not detectable in stereocilia (Ishibashi et al., 2008; Wang et al., 2019). A study by Ishibashi et al. (2009) demonstrated that after gentamicin treatment, the immunofluorescent reaction intensity for TRPV1 and TRPV2 in the mouse inner ear increased, whereas that for TRPV3 and TRPV4 decreased. This suggests that TRPV1 and TRPV2 may hold pathological significance for sensory cells and ganglions, while TRPV3 and TRPV 4 might play a crucial role in the neuroprotection of the inner ear.

Research on TRPV3 knockout mice has provided compelling evidence of its significance in auditory function. In the study by Wang et al. (2019), approximately 27.7% of TRPV3 knockout mice aged 2–3 months developed hearing impairment, in stark contrast to only 6% of wild-type mice. This substantial difference clearly indicates a crucial protective role of TRPV3 in maintaining normal hearing. Moreover, the compensatory mechanism within the TRP family is an interesting aspect. In TRPV3 knockout mice, the expression of TRPV4 in hair cells is significantly upregulated, and this upregulation effectively protects against hair cell damage caused by ototoxic drugs (Wang et al., 2019). This compensatory process showcases the remarkable adaptability of the TRP channel family. It implies that when one member, in this case TRPV3, is absent, other members like TRPV4 can step in to preserve auditory function, highlighting the intricate and well-coordinated interplay among TRP channels in safeguarding hearing. However, the underlying molecular mechanisms of this compensatory effect remain poorly understood. It is unclear how the absence of TRPV3 triggers the upregulation of TRPV4, and what signaling pathways are involved in this process. Therefore, further in-depth research is needed to verify and clarify these mechanisms, which will contribute to a more comprehensive understanding of the role of TRP channels in auditory function.

Ishibashi et al. (2008) identified the expression of TRPV4 in various cells of the normal mouse inner ear. These cells included hair cells and supporting cells of the organ of Corti, as well as marginal cells of the stria vascularis. This discovery supports the notion that TRPV4 contributes to maintaining the structure and function of the cochlea. The role of TRPV4 in hearing was demonstrated by Tabuchi et al. Their study revealed that TRPV4 knockout mice were more susceptible to acoustic injury and developed delayed-onset hearing loss (Tabuchi et al., 2005). Another study by Oonk et al. (2014) in a Dutch family with spinal muscular atrophy caused by a mutation in TRPV4, found significant differences in the manifestation and severity of hearing loss among family members with the same TRPV4 mutation, suggesting that TRPV4 is essential for maintaining cochlear function under stressful conditions, such as acoustic injury. These studies highlight the critical role of TRPV4 in maintaining hearing. Abnormalities in the TRPV4 gene (mutations or knockout) lead to hearing loss and cochlear hair cell damage.

#### 2.2.2 TRPV4 in drug-induced hearing loss

TRPV4 in drug-induced hearing loss is likely complex. Ishibashi et al. (2009) found that after treating mice with gentamicin via intratympanic injection (5 mg), the intensity of the immunofluorescent reaction to TRPV4 in the mouse inner ear decreased. This suggests that gentamicin may impact inner ear cell function by altering TRPV4 expression. A reduction in TRPV4 expression might weaken its protective effect against gentamicin-induced ototoxicity.

Conversely, Lee et al. (2013) demonstrated that in TRPV4 is involved in gentamicin-induced hearing loss at high dose (300mg/kg) by mediating the uptake of gentamicin. This suggests that TRPV4 can facilitate drug uptake, leading to hair cell damage and subsequent hearing impairment.

The reasons for the contradictory role of TRPV4 in drug-induced hearing loss are likely complex. It is evident that the function of TRPV4 is highly context-dependent. In the context of acoustic injury and delayed-onset hearing loss, TRPV4 seems to protect the cochlea, potentially by maintaining the integrity of cochlear structures or cellular functions. However, in the case of gentamicin-induced ototoxicity, the drug concentration can significantly affect TRPV4 function. Specifically, at low concentrations (e.g., 5 mg gentamicin), down-regulation of TRPV4 may help to reduce drug toxicity by decreasing TRPV4-mediated calcium influx, thereby lowering intracellular calcium levels and preventing calcium overload and subsequent cell damage. In contrast, at high concentrations (e.g., 300 mg/kg gentamicin) may over-activate TRPV4, resulting in calcium overload and cell damage (Lee et al., 2013). This concentration-dependent mechanism reveals the complex role of TRPV4 in ototoxicity.

Currently, although we have some insights into the roles of TRPV3 and TRPV4 in auditory function and their responses to ototoxic factors, research on their underlying molecular mechanisms is severely limited. For TRPV3, while we know it is crucial for maintaining normal hearing and its absence can trigger compensatory up-regulation of TRPV4, we lack knowledge about the specific upstream and downstream molecules in the signaling cascade responsible for this compensatory effect. Additionally, there is a dearth of research on how TRPV3 interacts with other proteins in cochlear cells to carry out its functions and what factors can modulate its activity.

Regarding TRPV4, its context-dependent function adds an extra layer of complexity. We need to conduct in-depth research to clarify how different cellular environments and physiological states precisely regulate TRPV4. This includes understanding the molecular mechanisms through which TRPV4 senses different stimuli and switches between its protective and potentially harmful roles. Research should also aim to identify the specific upstream and downstream molecules involved in TRPV4 signaling pathways. Understanding these interactions will provide insights into how TRPV4 modulates cellular responses to ototoxic agents and how it can be targeted therapeutically.

### 2.3 TRPV5, TRPV6, and hearing loss

#### 2.3.1 Structural features and expression patterns of TRPV5/TRPV6

In the TRPV family, TRPV5 and TRPV6 are structurally similar and both exhibit high selectivity for calcium ions (Rohacs et al., 2022). These channels are widely expressed in the cochlea and vestibule of mice. TRPV5 is primarily expressed in the marginal cells of the stria vascularis, vestibular sensory cells and dark cells, and less intensely in the spiral and vestibular ganglion cells, while TRPV6 is stably expressed in the supporting cells (Yamauchi et al., 2010; Takumida and Anniko, 2009; Takumida et al., 2009).

#### 2.3.2 Regulation by klotho and role in age-related hearing loss

Beyond their structural characteristics and expression profiles, the functional interplay between TRPV5/TRPV6 and regulatory molecules like klotho has become a focal point in understanding their role in age-related hearing loss, bridging basic channel biology to pathological mechanisms in auditory aging. Studies have shown that TRPV5 is regulated by the anti-aging protein klotho (Chang et al., 2005; Leunissen et al., 2013). Klotho can upregulate TRPV5 from both the inside and outside of cells by removing sialic acids from N-glycan of the channel and inhibiting its endocytosis exteriorly or likely enhancing forward trafficking of TRPV5 channel protein (Wolf et al., 2014). Age-related declines in klotho expression correlate with reduced TRPV5/TRPV6 levels in the inner ear. Takumida et al. (2009) found that, compared to 2-month-old mice, the expression of klotho in the cochlea was downregulated in 24-month-old mice, downregulation of klotho also leads to downregulation of TRPV5 and TRPV6, resulting in modified Ca^2+^ homeostasis in the inner ear, their downregulation in the aging inner ear may lead to auditory dysfunction.

#### 2.3.3 Functional insights and unresolved questions

Despite their calcium-regulatory roles, the direct involvement of TRPV5/TRPV6 in auditory mechanotransduction remains debated. In the M527C-Trpv6 knock-in mouse model (engineered for MTS reagent sensitivity), TRPV6 was shown non-essential for mechanotransduction, as MTS exposure failed to inhibit hair cell mechanoelectrical currents (Morgan et al., 2018). This highlights a gap in understanding: while TRPV5/TRPV6 are linked to calcium homeostasis and age-related hearing loss, their precise mechanistic roles (e.g., in sensory transduction vs. cellular calcium signaling) require further investigation.

## 3 TRPML channels in hearing loss

### 3.1 TRPML channels in cochlea

TRPML channels, characterized by a luminal/extracellular domain between transmembrane segments S1 and S2 (Venkatachalam and Montell, 2007b), are integral to lysosomal function, regulating processes such as autophagy and ion homeostasis (Zeevi et al., 2010; Rosato et al., 2021). Their expression in cochlear hair cells, marginal cells, and spiral ganglion neurons underscores their potential role in auditory physiology (Takumida and Anniko, 2010; Castiglioni et al., 2011).

### 3.2 TRPML3 dysfunction and hearing impairment

Given the integral role of TRPML3 in lysosomal function and its regulation of autophagy and ion homeostasis, understanding how its dysfunction leads to hearing impairment is pivotal for elucidating the molecular mechanisms underlying auditory pathologies. The varitint-waddler (Va) mouse model, carrying a constitutively active TRPML3 (A419P) mutation exhibits profound hearing loss linked to aberrant hair cell depolarization and ciliary defects (Palma et al., 2002; Nagata et al., 2008). Continuous ion influx through hyperactive TRPML3 channels induces calcium overload, triggering hair cell apoptosis (Grimm et al., 2007). This contrasts starkly with TRPML3 knockout mice, which show no significant auditory or vestibular deficits (Jörs et al., 2010). This paradox suggests that TRPML3 is dispensable under physiological conditions, but its gain-of-function mutation disrupts lysosomal-calcium dynamics, overwhelming compensatory mechanisms.

### 3.3 Synergistic roles of TRPML1 and TRPML3

While single deletion of TRPML1 or TRPML3 is non-pathogenic, combined knockout mice develop early-onset, progressive hearing loss (Wiwatpanit et al., 2018). At 4 months, these mice exhibit elevated auditory thresholds (12-27 kHz) and reduced hair cell survival, correlating with lysosomal swelling and permeabilization in outer hair cells. This indicates functional redundancy between TRPML1 and TRPML3 in maintaining lysosomal integrity, a critical safeguard against age-related cochlear degeneration.

Overall, the current research on TRPML channels and hearing loss has uncovered important relationships, but also raised many questions. Future studies should aim to further clarify the roles of individual TRPML channels, the nature of compensatory mechanisms, and the complex interplay between these channels and other cellular components in the context of auditory and vestibular function.

## 4 TRPC channels and hearing loss

TRPC channels have a TRP domain distal to the S6 Segment, containing TRP box 1 and TRP box 2 (Venkatachalam and Montell, 2007b), plays a crucial role in receptor-operated calcium entry, a process essential for the development of cardiac hypertrophy and heart failure (Beech, 2007). TRPC channels can be activated by G protein-coupled receptor (GPCR)-phospholipase C-diacylglycerol second messenger signaling and by a decrease in cytosolic Ca^2+^ concentration (Wong et al., 2013). TRPC channels are widely expressed in the mouse inner ear, including spiral ganglion neurons, the stria vascularis, and the vestibule (Takumida and Anniko, 2009). Given their wide expression in the mouse inner ear, it is reasonable to hypothesize that TRPC channels may also play an important role in auditory functions.

### 4.1 TRPC3 and auditoruy function

The high expression of TRPC3 in spiral ganglion neurons and inner and outer hair cells emphasizes its possible central role in auditory processes (Tadros et al., 2010), immediately suggests their potential importance in inner ear physiology. The study by Phan et al. (2010) on the temporal and spatial characteristics of TRPC3 expression in the mouse cochlea is a significant contribution. The observation that TRPC3 expression in embryonic hair cells remains high until 2 weeks after birth, and its expression in spiral neurons increases from the third week after birth, provides valuable insights into the role of TRPC3 during cochlear development. The conclusion that TRPC3 is involved in cochlear development and the formation of hearing is well-founded based on these findings. However, it is important to note that while the expression patterns are suggestive, more in-depth functional studies are needed to fully elucidate the exact mechanisms by which TRPC3 contributes to these processes. For example, genetic manipulation experiments during different stages of cochlear development could help clarify the causal relationship between TRPC3 expression and cochlear development.

The role of TRPC channels in regulating calcium ion influx in hair cells, as reported by Raybould et al. (2007) is a fundamental aspect of their function. Maintaining intracellular calcium homeostasis is crucial for numerous cellular processes, and in the context of hair cells, it is essential for auditory nerve conduction. The presence of TRPC3 in inner and outer hair cells and its role in this calcium-regulating mechanism is an important piece of the puzzle.

The findings from TRPC3 knockout mice studies by Wong et al. (2013) are particularly interesting. The enhanced outer hair cell electromotility and increased hearing sensitivity, especially in the 8–16 kHz range, in TRPC3 knockout mice suggest that TRPC3 has a regulatory function in normal hair cell membrane conductance and outer hair cell function. However, this also raises questions about the complex compensatory mechanisms at play. In the absence of TRPC3, other channels or proteins may be upregulated or modified to compensate for its loss, leading to these changes in hair cell function.

Following the exploration of TRPC3's specific role in auditory function, the subsequent section expands to examine the cumulative effects of combined TRPC channel knockout in hearing loss, bridging single-channel mechanisms to broader functional insights into their collaborative roles in auditory pathology.

### 4.2 Combined knockout of TRPC channels in hearing loss

The findings regarding the double knockout of TRPC3 and TRPC6 are significant (Quick et al., 2012). The fact that single-knockout mice display normal behavioral phenotypes while double-knockout mice exhibit hearing loss and vestibular deficits indicates that these two channels may have overlapping or compensatory functions. It's possible that when both are absent, the normal physiological processes in the inner ear related to hearing and balance are disrupted beyond the capacity of other channels or proteins to compensate. However, the exact mechanisms by which the loss of these two channels leads to such deficits remain unclear. Are they involved in the same signaling pathways, or do they regulate different aspects of inner ear cell function that are both essential for normal auditory and vestibular function? Future studies could explore these questions through in-depth molecular and cellular analyses.

The results from the quadruple knockout of TRPC1, TRPC3, TRPC5, and TRPC6 by Sexton et al. (2016) further expand our understanding. The significant impairments in tactile sensitivity and hearing function, along with elevated auditory thresholds at high frequencies, suggest that these channels play important roles in sensory perception, particularly in the auditory system. The lack of difference in mechanoelectrical transducer currents in outer hair cells between knockout and wild-type mice, however, is a surprising finding. This challenges the initial assumption that these channels might be candidates for mechanoelectrical transduction channels in hair cells.

It's important to consider the limitations of these knockout models. The knockout of multiple channels simultaneously might trigger complex compensatory responses that could mask the true functions of individual channels. For example, the absence of one channel could lead to the upregulation or altered function of other channels, which might compensate for the loss in some aspects but not others.

Moreover, the fact that these channels are expressed in various cell types in the inner ear, including spiral ganglion neurons and hair cells, suggests that their functions could be diverse. They might be involved in processes other than mechanoelectrical transduction, such as maintaining the proper physiological environment for hair cell function, regulating neurotransmitter release in the auditory pathway, or contributing to the development and maturation of the inner ear. While these studies have provided valuable insights into the role of TRPC channels in auditory and vestibular function, there is still much to be learned.

### 4.3 TRPC channels expression in the cochlea of humans

The discovery by Englisch et al. (2023) of TRPC expression in the human cochlea represents a significant advancement in the field of auditory research. By using 3D CT reconstruction technology to precisely locate the cochlea in human specimens, followed by paraffin sectioning and immunohistochemical staining, the researchers were able to identify the presence of TRPC3 and TRPC6 in the spiral neurons of the human cochlea, with TRPC6 also detected in the organ of Corti. This is a crucial step in understanding the potential role of TRPC channels in human auditory function, as previous studies had mainly focused on animal models.

The identification of TRPC expression in the human cochlea provides a solid foundation for further exploration of the relationship between these channels and hearing. The fact that TRPC3 and TRPC6 are expressed in key structures involved in auditory signal transduction, such as spiral neurons and the organ of Corti, strongly suggests that they could play a role in hearing formation. However, at this stage, the nature of their involvement remains speculative.

One of the study's key strengths lies in its utilization of human specimens. This approach directly addresses the translational significance of TRPC channels in human hearing. In contrast to animal-based research, where species-specific disparities can impede the direct application of findings to humans, Englisch et al. have made a notable advancement in our comprehension of TRPC channels within the human cochlea. However, it should be noted that the study solely reports on the presence of TRPC3 and TRPC6, leaving the expression patterns of other TRPC family members in the human cochlea undetermined. Additionally, the relatively small sample size of human specimens used in this study may limit the generalizability of the findings, potentially overlooking rare or variable expression patterns that could exist in a larger population.

## 5 TRPM and hearing loss

The TRPM subfamily, which includes TRPM1 through TRPM8, exhibits diverse functional properties and is involved in a wide range of cellular processes, including cell proliferation and cell death (Watanabe et al., 2008).

### 5.1 TRPM1 and auditory function

Studies by Gerka-Stuyt et al. (2013) and Bellone et al. (2008) collectively demonstrate that TRPM1 is dispensable for auditory and vestibular function in mice. Hearing thresholds and auditory brainstem response (ABR) waveforms in Trpm1^−^/^−^ and Trpm1^tvrm27/tvrm27^ mice showed no statistically significant differences compared to littermate controls across tested frequencies. Vestibular behavior (e.g., circling, head bobbing) remained normal in Trpm1^−^/^−^ mice, indicating preserved balance function. These findings suggest that TRPM1 is not essential for hearing or balance in mice and is unlikely to be a component of the hair cell mechanotransduction (MET) channel (Gerka-Stuyt et al., 2013). These findings exclude TRPM1 as a critical component of the hair cell mechanotransduction (MET) channel. Mutations in TRPM1 are associated with congenital stationary night blindness in humans and depolarizing bipolar cell dysfunction in mouse retinas, yet no auditory phenotypes are observed, reinforcing its non-essential role in hearing (Bellone et al., 2008).

### 5.2 TRPM2 and hearing loss

The TRPM2 channel can be activated by intracellular ADP-ribose (ADPR), NAD, and reactive oxygen/nitrogen species (Kühn and Lückhoff, 2004). Compelling evidence from molecular, immunohistochemical, and electrophysiological studies demonstrates the expression of functional TRPM2 channels in the stellate neurons of the mouse ventral cochlear nucleus (VCN). Oxidative stress, a well-established risk factor for idiopathic sudden sensorineural hearing loss, noise-induced hearing loss and age-related hearing loss, is capable of triggering TRPM2 channel activation. Once activated, TRPM2 causes the resting membrane potential of neurons to shift toward depolarization, which may play a role in the development of these auditory pathologies (Bal et al., 2020).

While this sequence of events, from oxidative stress to TRPM2 activation and neuronal depolarization, offers a potential mechanistic explanation for a common and often disabling auditory disorder, direct experimental validation remains limited, and no studies have yet definitively linked TRPM2 dysfunction to specific auditory phenotypes *in vivo*. This limitation underscores the need for more robust *in vivo* models to elucidate the role of TRPM2 in auditory processes and pathologies. In addition, while the activation mechanisms of TRPM2 are well-characterized, the precise downstream effects and interactions with other cellular pathways in the context of auditory function are not fully understood. This includes the molecular mechanisms through which TRPM2-mediated depolarization contributes to hearing loss.

Thus, it is needed to develop and utilize more sophisticated *in vivo* models, such as genetically modified mice with targeted TRPM2 knockouts or overexpressions, to study the effects of TRPM2 on auditory function and pathologies. It is also needed to investigate the downstream signaling pathways activated by TRPM2 in auditory neurons. This includes understanding how TRPM2-mediated calcium influx and depolarization lead to cellular changes that contribute to hearing loss. Additionally, techniques such as single-cell RNA sequencing and proteomics can provide insights into the molecular networks involved. It is also needed to conduct functional assays to assess the impact of TRPM2 activation on auditory processing, including electrophysiological recordings, behavioral hearing tests, and imaging techniques to evaluate changes in auditory function and neural activity in response to TRPM2 modulation.

### 5.3 TRPM4 and auditory function

#### 5.3.1 Functional properties and regulatory mechanisms

TRPM4 plays a significant role in modulating various calcium-dependent mechanisms within cells. The interaction of its N-terminus region with phosphoinositide lipids (PIPs), such as PIP2 and PIP3, as discovered by Bousova et al. (2015), adds a layer of complexity to its regulatory function. This interaction likely fine-tunes the channel's activity, influencing the flow of ions and ultimately impacting cellular processes that rely on calcium signaling.

#### 5.3.2 Spatiotemporal expression of TRPM4 in the auditory system

Sakuraba et al.'s (2014) study on the temporal and spatial expression of TRPM4 in the mouse cochlea provides valuable insights. The specific expression pattern of TRPM4 in the apical side marginal cells of the stria vascularis, in the cell body of inner hair cells, and in a subset of the type II neurons in the spiral ganglion indicates its potential involvement in the normal functioning of the cochlea. These cell types are crucial for the transduction of sound signals into neural impulses, suggesting that TRPM4 may contribute to the auditory process at a fundamental level. TRPM4 expression is weak from birth (P0) to 1 week after birth (P7) in inner hair cells and spiral neurons, followed by a significant upregulation after birth (P14) (Sakuraba et al., 2014). This upregulation coincides with the onset of hearing in mice, suggesting that TRPM4 may be involved in the maturation of the auditory system.

TRPM4 is also expressed in non-sensory hair cells of the crista ampullaris and dark cells within the anterior semicircular canal, implicating a potential role in balance regulation (Sakuraba et al., 2014). However, its exact function in vestibular mechanotransduction or endolymph secretion remains uncharacterized.

The developmental expression pattern of TRPM4 in the cochlea aligns with the onset of hearing in mice (Shnerson and Pujol, 1981). This correlation strongly suggests that TRPM4 may be involved in the initiation of the hearing process. It could potentially be involved in the maturation of the auditory system, perhaps by regulating calcium-dependent processes that are essential for the proper functioning of inner hair cells and spiral ganglion neurons as they start to respond to sound stimuli.

However, it is important to note that while this correlation is suggestive, it does not prove a causal relationship. There could be other factors at play during the development of hearing in mice, and TRPM4′s upregulation may be a secondary effect or part of a larger regulatory network. Future studies should aim to directly manipulate TRPM4 expression or function during this critical developmental period to determine its exact role in the onset of hearing. This could involve genetic knockout or knockdown experiments in mice, followed by detailed auditory function assessments. Overall, the current findings on TRPM4's expression in the mouse cochlea open up new avenues for research into the molecular mechanisms underlying hearing development.

### 5.4 TRPM6 and TRPM7 in auditory function

The co-immunoprecipitation assays in HEK cells, which showed interactions between TRPM6, TRPM7, and key hair-cell proteins like USH1C (harmonin) and PCDH15, initially suggested a promising connection to the transduction complex (Morgan et al., 2018). The experiments painted a more complex picture. Manipulations of intracellular Mg^2+^, which are known to influence the function of TRPM6 and TRPM7, failed to impact hair-cell transduction currents (Zou et al., 2019). This lack of effect was unexpected, considering the established link between Mg^2+^ and the function of these channels. It suggests that, despite the known regulatory role of Mg^2+^ on TRPM6 and TRPM7, the channels' function might not be as directly related to hair-cell transduction currents as initially hypothesized. This could imply that the channels have alternative functions or that the relationship between Mg^2+^ regulation and hair-cell transduction is more complex than previously thought.

The results from the conditional knockout mice further complicated the understanding of TRPM6 and TRPM7′s role in auditory function. The fact that conditional deletion of these channels in hair cells did not affect auditory function, as measured by ABR measurements, strongly suggests that TRPM6 and TRPM7 do not play a significant role in hair-cell mechanotransduction (Morgan et al., 2018). This finding challenges the initial hypothesis based on the co-immunoprecipitation results.

It's important to note that these negative results should be interpreted with caution. The conditional knockout approach has its limitations. There could be compensatory mechanisms at play. Other proteins or channels in the hair cells might be able to take over the functions that TRPM6 and TRPM7 would otherwise perform. Additionally, the lack of effect on ABR measurements might not necessarily mean that these channels have no role in hair-cell function at all. They could be involved in other, more subtle aspects of auditory function that were not captured by the ABR assay.

## 6 TRPA1 and hearing loss

TRPA1 is a typical member of this subfamily, characterized by 14 ankyrin repeat sequences in the N-terminal domain (Venkatachalam and Montell, 2007b). The initial identification of TRPA1 as a channel expressed in outer hair cells by Corey et al. (2004) was a significant discovery, suggesting its potential involvement in the electromechanical transduction of hearing. This hypothesis was plausible considering the significant role that outer hair cells play in auditory function. Nevertheless, the subsequent discovery by Kwan et al. (2006), which revealed that TRPA1 homozygous knockout mice showed no hearing abnormalities, contradicted this initial presumption. Such discrepancies are not uncommon in scientific research and highlight the importance of multiple lines of evidence. This could be due to compensatory mechanisms in the knockout mice, where other channels or proteins may have taken over the functions that TRPA1 was initially thought to perform.

Although knockout of TRPA1 has no impact on hearing, it can mediate the accumulation of aminoglycosides in the outer hair cells of the mouse cochlea (Stepanyan et al., 2011). This finding indicates a potential role of TRPA1 in drug-induced hearing loss. Nevertheless, further verification through animal studies is required.

Study by Vélez-Ortega et al. (2023) have identified TRPA1 expression in non-sensory supporting cells of the mouse cochlea, including Hensen cells, Deiters cells, and pillar cells. This discovery has opened new research directions. They found that when TRPA1 is activated in Hensen cells, it triggers prolonged Ca^2+^ responses. These responses propagate across the organ of Corti, resulting in long-lasting contractions of pillar and Deiters' cells. After noise exposure, TRPA1 activation in these supporting cells may impact cochlear sensitivity. For instance, TRPA1 deficiency leads to larger yet less prolonged noise-induced temporary shifts in hearing thresholds, along with permanent changes in the latency of the ABR.

Overall, identifying TRPA1 activation in these non-sensory supporting cells and understanding its role in regulating cochlear sensitivity following acoustic trauma represents a significant advancement. The mechanism by which TRPA1 activation in Hensen cells causes Ca^2+^ responses that lead to contractions of pillar and Deiters cells offers a potential explanation for how TRPA1 can affect cochlear function.

The fact that TRPA1 can be activated by endogenous products of oxidative stress and extracellular ATP, both of which are present after acoustic trauma, further emphasizes its role in the cochlear response to injury (Vélez-Ortega et al., 2023). This suggests that TRPA1 may act as a sensor for cochlear stress and initiate a series of cellular events to protect the cochlea. The observation that TRPA1-deficient mice exhibit larger but less prolonged temporary shifts in hearing thresholds after noise exposure, along with permanent changes in the latency of auditory brainstem responses, supports the idea that TRPA1 plays an important role in regulating cochlear sensitivity after acoustic trauma.

However, there is still much to be learned. For example, the exact signaling pathways downstream of TRPA1 activation in non-sensory supporting cells are not fully understood. It is unclear how the function of TRPA1 in non-sensory supporting cells interacts with other cochlear cell types, such as hair cells and spiral ganglion neurons.

## 7 Discussion

This review offers a comprehensive overview of the diverse roles played by TRP channels in auditory function and hearing loss. Their participation in multiple forms of hearing loss, including those induced by drugs, noise, and age-related degeneration (Table 1), highlights their potential as promising therapeutic targets.

The complexity inherent in the functions of TRP channels is quite apparent. Each subfamily and even individual channel members exhibit distinct behaviors that are often contingent upon the cellular context. The context-dependent functions and the compensatory mechanisms witnessed not only underscore the intricacy of these channels but also emphasize the necessity for further in-depth research to fully clarify their underlying action mechanisms. Future research endeavors should concentrate on the development of highly specific and effective TRP channel modulators. Additionally, exploring the interactions between TRP channels and other cellular pathways involved in auditory function is crucial for a more comprehensive understanding of the auditory system.

Furthermore, the translational potential of findings from animal studies to human applications represents a critical aspect in this field. Although numerous studies on TRP channels have been carried out using animal models, the biological differences between species can impede the direct extrapolation of these results to humans. For instance, while the discovery of TRPC3 and TRPC6 expression in the human cochlea is a significant milestone, additional research is required to confirm whether the functions observed in animal models are applicable to humans. Moreover, the relatively small sample sizes commonly used in human studies may result in overlooked expression patterns or functional discrepancies. Therefore, large-scale studies, in conjunction with advanced techniques for examining human cochlear function, are indispensable for bridging the gap between basic research and clinical applications in the treatment of hearing loss.

Future research should also delve into the mechanisms underlying TRP channel function in hearing loss, particularly focusing on the roles of mitochondria and inflammation. Mitochondrial dysfunction, which can lead to imbalances in intracellular calcium ion homeostasis, is increasingly recognized as a key factor in hearing loss, thereby potentially contributing to hair cell damage. Understanding the interplay between TRP channels and mitochondrial dynamics could provide new insights into the pathophysiology of hearing loss. Inflammation is another critical factor in hearing loss, particularly in drug-induced and noise-induced damage. TRP channels have been shown to mediate inflammatory responses by regulating the release of pro-inflammatory cytokines and reactive oxygen species (ROS). In addition, TRP channels are expressed on circulating and resident immune cells. However, whether they are activated by cochlear stressors to initiate cochlear inflammation and ototoxicity needs to be determined. Future research should explore how TRP channels interact with inflammatory pathways and how these interactions contribute to hearing loss. What's more, existing research has confirmed the expression of the TRPP subtype in the cochlea, where the expression of TRPP2, TRPP3, and TRPP5 in cochlear spiral ganglion neurons has been detected (Takumida and Anniko, 2010). However, there are currently very few studies to reveal their roles in auditory function. Further research is needed in the future to explore the contribution of TRPP channels to auditory function.

As our knowledge of TRP channels continues to expand, we expect to see remarkable progress in the development of innovative therapeutic strategies for hearing loss and related auditory disorders. This advancement holds the promise of improving the quality of life for countless individuals affected by these conditions.
